# Sunitinib Combined with Th1 Cytokines Potentiates Apoptosis in Human Breast Cancer Cells and Suppresses Tumor Growth in a Murine Model of HER-2^pos^ Breast Cancer

**DOI:** 10.1155/2021/8818393

**Published:** 2021-04-14

**Authors:** Nirmala Ghimirey, Chase Steele, Brian J. Czerniecki, Gary K. Koski, Loral E. Showalter

**Affiliations:** ^1^Department of Biological Sciences, Kent State University, Kent, Ohio 44242, USA; ^2^Department of Breast Oncology, H. Lee Moffitt Cancer Center, Tampa Florida 33612, USA

## Abstract

Although immune-based therapies have made remarkable inroads in cancer treatment, they usually must be combined with standard treatment modalities, including cytotoxic drugs, to achieve maximal clinical benefits. As immunotherapies are further advanced and refined, considerable efforts will be required to identify combination therapies that will maximize clinical responses while simultaneously decreasing the unpleasant and sometimes life-threatening side effects of standard therapy. Over the last two decades, evidence has emerged that Th1 cytokines can play a central role in protective antitumor immunity and that combinations of Th1 cytokines can induce senescence and apoptosis in cancer cells. To explore the possibility of combining targeted drugs with Th1-polarizing vaccines, we undertook a study to examine the impact of combining Th1 cytokines with the relatively broad-spectrum receptor tyrosine kinase antagonist, sunitinib. We found that when a panel of five phenotypically diverse human breast cancer cell lines was subjected to treatment with sunitinib plus recombinant Th1 cytokines IFN-*γ* and TNF-*α*, synergistic effects were observed across a number of parameters including different aspects of apoptotic cell death. Interestingly, sunitinib was found to have a profoundly suppressive effect of T cell's capacity to secrete IFN-*γ*, indicating that in vivo use of this drug may hinder robust Th1 responses. Nonetheless, this suppression was circumvented in a mouse model of HER-2^pos^ breast disease by supplying recombinant interferon-gamma to achieve a combination therapy significantly more potent than either agent.

## 1. Introduction

For HER-2^pos^ breast malignancies, better clinical outcomes are observed when CD4^pos^ lymphocytic infiltrates display characteristics of Th1-type cells [1]. Interestingly, DC-based neoadjuvant vaccines that selectively promote the development of strong anti-HER-2 Th1 immunity, defined by CD4 T cell secretion of select cytokines such as IFN-*γ* and TNF-*α*, can induce peritumoral accumulation of CD4^pos^ (helper T cells) and CD20^pos^ (B cells) lymphocytes at the site of HER-2^pos^ early breast cancer (ductal carcinoma in situ, DCIS) [2, 3]. In these studies, around 30% of vaccinated HER-2^pos^ ER^neg^ subjects had complete pathological responses (defined as no detectable remaining disease) at the time of surgery. For ER^pos^ subjects, only 5% showed pCR. In vitro studies demonstrated that the principle Th1 cytokines could induce senescence and also apoptosis in various HER-2^pos^ and HER-2^neg^ breast cancer lines [4, 5].

Interestingly, some ER^pos^ breast cancer lines showed resistance to Th1 cytokine-induced apoptosis, but when antiestrogen drugs were combined with cytokines, the cells showed apoptosis levels consistent with their ER^neg^ counterparts [6]. These studies seemed to indicate that pharmacological inhibition of growth factor signaling (in this case through ER receptor) could sensitize previously resistant cells to cytokine-induced cell death. This finding suggested a clinical trial where antiestrogen drugs would be supplied coincident with vaccination for HER-2^pos^/ER^pos^ subjects. Here, it was found that antiestrogen therapy plus vaccination equalized pCR rates of ER^pos^ subjects with ER^neg^ vaccine recipients at around 30% [6].

Such studies suggest that pharmacological inhibition of growth factor signaling combined with vaccine-induced generation of Th1 immunity can enhance vaccine response rates. They also suggest that Th1 cytokines can be used as an in vitro surrogate for Th1 immunity to screen candidate drugs for combined antitumor effects to select promising agents for testing in clinical trials.

Unlike estrogen receptor, most growth factor receptors are receptor tyrosine kinases. In order to test the hypothesis that inhibition of growth factor signaling is a good overall strategy for improving Th1 cytokine-mediated antitumor immunity, we investigated the capacity of a broad-spectrum RTK antagonist drug, sunitinib, in combination with Th1 cytokines, to work cooperatively to enhance killing activity against breast carcinoma cells. Sunitinib malate is an orally administered, small-molecule inhibitor that interferes with the activity of multiple RTKs including VEGFR, KIT, FLT3, and PDGFR. *In vitro* studies have demonstrated that sunitinib inhibits the growth of human umbilical vein endothelial cells that are dependent on VEGF, PDGF, and stem-cell factor (SCF). Sunitinib demonstrated both time- and dose-depended antitumor activities in human tumor xenograft models [7] for colon [8], glioma, melanoma, and breast [7] cancer cell lines. It has also been tested in a clinical trial for breast cancer patients and has been examined in combinations with other drugs such as trastuzumab [9], docetaxel [10], and capecitabine [11]. And although the drug did not show sufficient activity against breast cancer to warrant adoption as part of a standard-of-care treatment regimen for this malignancy, its current indications do include gastrointestinal stromal tumors, advanced renal cell carcinoma, and some pancreatic neuroendocrine tumors.

In the studies presented here, we show that the combination of sunitinib and Th1 cytokines can lead to greatly enhanced activity against a panel of 5 human breast cancer cell lines representing a variety of distinct phenotypes, suggesting that an overall strategy of interference with receptor tyrosine kinases in conjunction with effectors of Th1 immunity may represent a useful approach for developing improved immunotherapy. A potential caveat uncovered was the tendency of sunitinib to also profoundly inhibit IFN-*γ* secretion by T lymphocytes. Successful development of this general approach will probably involve the use of more selective RTK antagonists that do not strongly impact T cell function. Alternatively, recombinant T cell effector cytokines along with drug can be administered so that therapeutic levels of both agents can be achieved even if T cells are unable to biosynthesize these molecules under drug pressure.

## 2. Materials and Methods

### 2.1. Reagents and Cell Culture

Sunitinib (Selleckchem, Houston, TX) was dissolved in dimethyl sulfoxide (DMSO; Sigma-Aldrich, St. Louis, MO) and diluted in cell culture media or PBS for in vitro use. Human breast cancer cell lines MDA-MB-231, MDA-MB-468, SKBR-3, MCF-7, and HCC-1419 were obtained from American Type Culture Collection (ATCC, Manassas, VA). The MDA-MB-231, HCC-1419, and MDA-MB-468 cell lines were cultured in RPMI media (Sigma-Aldrich). MCF-7 cell lines were cultured in EMEM media (ATCC). SKBR-3 cells were cultured in McCoy's 5A media (Gibo; Rockville, MD). In all media, 10% fetal bovine serum (Atlantic Biologicals, Miami, FL), 1% Pen/strep (Gibco), 1% sodium pyruvate, 1% NEAA, and 1% glutamine (Gibco) were added and cultured cells were maintained at 37°C and 5% CO_2_ humidified atmosphere.

### 2.2. Alamar Blue Assay

Breast cancer cell lines were plated in 96-well cluster plates at densities ranging from 5 to 7 × 10^3^ cells per well depending on the growth characteristics of individual lines. After 12 hours of initial culture, cells were treated with Th1 cytokines (TNF-*α* and IFN-*γ*; 5 ng/mL each PeproTech, Rocky Hill, NJ) and sunitinib (10 *μ*M), then cultured for 72 additional hours, at which time 20 *μ*L of 0.15 mg/mL of resazurin salt in PBS (Sigma-Aldrich) was added to each well. After 6-7 hours of additional culture, the optical density of the culture supernatants was measured at 630 nm using a Bio-Tek (Model EL312E) microplate reader using Gen5 analysis software.

### 2.3. Viability Staining with Trypan Blue

Breast cancer cells were seeded at a density of 5 × 10^4^/mL in 12-well cluster plates and incubated overnight. The next day, the cells were treated with cytokines (5 ng/mL each TNF-*α* and IFN-*γ*), sunitinib (10 *μ*M), both, or left untreated. After 72 hours of further incubation, cells were harvested by scraping, and 0.002% Trypan Blue dye (Lonza, Walkersville, MD) was added in 30 *μ*L volume of cell suspension. Dye uptake was assessed via flow cytometry using a FlowSight cytometer (Amnis/Millipore) employing a 642 nm laser, and analysis performed using IDEAS version 6.2 software as described previously [12, 13].

### 2.4. Annexin V/Propidium Iodide Staining

Breast cancer cell lines were treated and cultured as described for Trypan Blue studies. At 72 hours posttreatment, cells were harvested by scraping and washed with Annexin V staining buffer (BioLegend, San Diego, CA). Cells were then stained with fluorochrome-labeled Annexin V (3 *μ*L added in 40 *μ*L total volume) (BioLegend) at 4°C for 15 minutes, followed by the addition of propidium iodide (PI) (1 *μ*g/mL) (Sigma-Aldrich) immediately prior to analysis using a FlowSight imaging flow cytometer (employing the 642 nm and 488 nm lasers) and running IDEAS version 6.2 software as described previously [12, 13].

### 2.5. Assessment of Relative Levels of Mitochondrial Cytochrome C

SKBR-3 cell lines were treated as described for Trypan Blue studies. At 72 hours posttreatment, cells were harvested with the aid of trypsin (Gibco) and stained for the presence of intramitochondrial cytochrome C according to the method of Christensen et al. [14]. Briefly, cells were resuspended in 100 *μ*L ice-cold permeabilization buffer (100 mM KCL, 100 *μ*g/mL digitonin in PBS). These were incubated for 5 minutes, and 100 *μ*L paraformaldehyde (4% in PBS) was added to the permeabilized cells. Samples were centrifuged at 500 RPM at 4°C for 5 minutes followed by removal of the supernatant. Cells were then incubated with 4% paraformaldehyde for 20 minutes at room temperature. Cells were then washed three times in 200 *μ*L PBS (700 RPM for 5 min at 4°C) and resuspended in 200 *μ*L blocking buffer (0.05% saponin and 3% BSA in PBS), then incubated for 1 hour at RT. APC-conjugated anti-cytochrome C (1 : 200) (BioLegend) was added and incubated overnight at 4°C. The next day, cells were washed three times in 200 *μ*L PBS (700 RPM for 5 min at 4°C). Anti-mouse secondary (1 : 200) antibody (BioLegend) was added, diluted in 100 *μ*L blocking buffer, and incubated for an hour at RT. Cells were washed three more times in 200 *μ*L PBS (700 RPM for 5 min at 4°C). The expression of cytochrome C was analyzed using a FlowSight imaging cytometer employing 642 nm and IDEAS software suite version 6.2.

### 2.6. Assessment of HER-2 Surface Expression

SKBR-3 cells were treated as described for Trypan Blue studies. At 72 hours posttreatment, cells were harvested by scraping, washed in PBS, and resuspended in 1 mL FACS buffer (PBS+1%FBS+0.01% sodium azide). APC-conjugated anti-HER-2 antibody (BioLegend, San Diego, CA) was then added for 15 minutes at 4°C, and cells were analyzed for HER-2 expression by flow cytometry using 642 nm excitation laser on a FlowSight imaging cytometer running IDEAS 6.2 analysis software.

### 2.7. Assessment of PARP Status via Western Blot Analysis

SKBR-3 cells were seeded at 3 × 10^6^ cells/mL in 10 mL total volume in a petri dish. The next day, cells were treated with 5 ng/mL of Th1 cytokines, 10 *μ*M sunitinib, or both. After incubation for 72 hours, samples were collected. Harvested cells were centrifuged at 800 RPM for 5 minutes, and supernatants were removed and discarded. The cell pellets were lysed using 100 *μ*L of RIPA lysis buffer (50 mM Tris-base, 150 mM NaCl, 1% Triton-X 100, 0.5% sodium deoxycholate, and 0.1% sodium dodecyl sulfate) with added Pierce protease inhibitor cocktail (Thermo Scientific, Rockford, IL) and PhosStop phosphatase inhibitor cocktail (Roche, Mannheim, Germany). These extracts were centrifuged at 20,000 RPM for 20 minutes at 4°C. Supernatants were collected and stored at -80°C prior to analysis.

The BCA protein assay was used to generate a standard curve to estimate the amount of proteins in each extract. Extracts were diluted in 5x reducing loading buffer, vortexed briefly, and boiled for 5 minutes. Thirty micrograms of total protein from each sample were loaded into 4-15% Mini Protean TGX gels (Bio-Rad, Hercules, CA) and separated for 40 minutes at 160 volts. Proteins from the gel were electrotransferred onto PVDF membrane (Bio-Rad) at 100 volts for an hour. Membranes were washed 3 times with TBS-T (20 mM Tris, 150 mM NaCl, and 0.1%Tween 20 with pH of 7.6) at 5-minute intervals. The membranes were blocked with 5% milk for 30 minutes and incubated overnight with primary antibodies: 1 : 1000 PARP anti-rabbit (Cell Signaling, Denver, MA) and 1 : 1000 anti-*β*-actin anti-mouse (GenScript, Piscataway, NJ). The membrane was washed with TBS-T buffer, and an appropriate secondary HRP-tagged antibody was added and incubated at room temperature for an hour. This was followed by washing the membrane 3 times with TBS-T at 5-minute intervals. The membrane was developed by using SuperSignal West Pico Chemiluminescent Substrate (Thermo Scientific). Chemiluminescent detection was performed using the Fuji LAS 3000 detection system (R&D Systems, Minneapolis, MN). Band intensity and fold change were quantified using ImageJ analysis.

### 2.8. T Cell Functional Assays

For allogeneic MLRs, human peripheral blood mononuclear cells were obtained from healthy volunteers via leukapheresis and used in experiments after provision of informed written consent, and in accordance with the principles of the Declaration of Helsinki and NIH guidelines for human subjects, through protocols approved by the Institutional Review Boards of the Cleveland Clinic (08–957) and Kent State University (18–421). Blood products were separated into CD14^pos^ peripheral blood monocyte and lymphocyte-enriched fractions via countercurrent centrifugal elutriation as described previously [15] and maintained cryopreserved in liquid nitrogen until use. Dendritic cells were derived from monocytes that were thawed and incubated overnight in Macrophage SFM media (Gibco) and supplemented with GM-CSF (50 ng/mL) (PeproTech) and IL-4 (1000 U) (PeproTech). The next morning, 1000 U of IFN-*γ* (PeproTech) was added to the culture, and after 2 additional hours, LPS (50 ng/mL; InvivoGen, San Diego, CA) was added. The cells were allowed to incubate for another 4 hours before being harvested, washed, and resuspended in RPMI supplemented with 5% human serum (Lonza). Allogeneic lymphocyte-enriched fractions were thawed and brought up in an identical culture medium. The cells were combined at a ratio of 20 : 1 (lymphocytes: dendritic cells) and plated in a 48-well cluster dish at 1 mL total volume per well. The allogeneic coculture was either treated with sunitinib (10 *μ*M) or left untreated for control. After 72 hours, the supernatants were collected and analyzed for IFN-*γ* via ELISA according to the manufacturer's protocol (BD Biosciences, San Diego, CA).

For ELISPOT, cryopreserved, unfractionated total peripheral blood mononuclear cells (PBMCs) obtained from normal healthy donors and IFN-*γ* ELISPOT kits were purchased from Cellular Technology Limited (C.T.L., Shaker Heights, OH). As per the manufacturer's recommendations, the PBMC's were thawed and plated at a density of 200,000 cells per well (either in the presence or absence of 10 *μ*M sunitinib) with CEF-Class I Peptide Pool “Plus” (a mixture of common viral peptides; 10 *μ*L/well, C.T.L) or tetanus toxin (2 *μ*g/well, Sigma) as recall antigens or incubated in media alone (control). The next day, the cells were removed and a number of IFN-*γ* spot-forming cells were assessed as per kit instructions. All plates were read on an ImmunoSpot analyzer (C.T.L).

### 2.9. Treatment Model

Female Balb/c mice, 6-8 weeks of age, were purchased from Charles River Laboratories (Wilmington, MA). They were maintained in accordance with the National Institute of Health's guidelines, in a specific pathogen-free environment in the animal facility in Cunningham Hall at Kent State University. Experiments were approved by the Institutional Animal Care and Use Committee (IACUC) of Kent State University under the protocol 481 GK-19-05. TUBO breast carcinoma cells, which were derived from Balb-neuT rat HER-2 (neu) transgenic mice (Rovero et al. 2000), were cultured in RPMI supplemented with 10% *v*/*v* fetal calf serum (FBS; Atlanta Biologicals, Flowery Branch, GA), 100 units/mL of penicillin and 100 *μ*g/mL of streptomycin sulfate (BioWhittaker, Walkersville, MD), 2 mmol/L glutamine (BioWhittaker), 1 mmol/L sodium pyruvate (BioWhittaker), and 1% nonessential amino acids (BioWhittaker). Cells were harvested with Cell Dissociation Buffer (Gibco, Grand Island, NY), washed 2 times with PBS, and then resuspended in PBS for inoculation.

Mice were divided into 4 groups containing 6 mice per group. On day 0, all mice were subcutaneously inoculated, into the region of the fat pad of the breast, with 2.5 × 10^5^ TUBO cells in 100 *μ*L of PBS. On day 7, when tumors were palpable, mice were either left untreated (control), treated via i.p. injection with sunitinib (1 mg/dose), IFN-*γ* (10 *μ*g), or both treatments simultaneously. Schedules of administration are indicated for individual experiments. Calipers were used to measure tumor size (length along the longest axis multiplied by perpendicular width) every 2-3 days.

### 2.10. Statistical Method

One-way Analysis of Variance (ANOVA) was performed for statistical analyses; *p* value of less than 0.05 was considered significant. The statistical significance between treatment groups was determined using Tukey's honestly significant difference test. One-way ANOVA was analyzed using SigmaPlot 12.5 software (Systat Software Inc., San Jose, CA).

## 3. Results

### 3.1. Th1 Cytokines and Sunitinib Work Together to Suppress Breast Cancer Cell Metabolism

**Figure 1:**
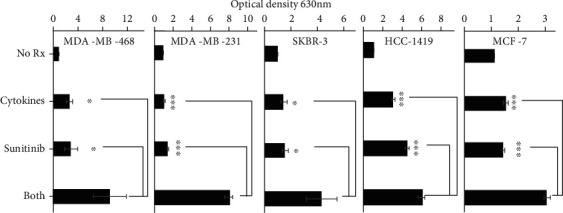
Metabolic activity of breast cancer cell lines is maximally suppressed by Th1 cytokines and sunitinib treatment. MDA-MB-468, MDA-MB-231, SKBR-3, HCC-1419, and MCF-7 cells were seeded at 5 × 10^3^ cells per well and were left either untreated (No Rx) or treated with Th1 cytokines (5 ng/mL each IFN-*γ* and TNF-*α*), sunitinib (10 *μ*M), or sunitinib and Th1 cytokines (both). After 72 hours of incubation, Alamar Blue dye was added to each well. When a color change became apparent, the optical density of the supernatants was read via spectrophotometry at 630 nm. Data represent the summary analysis of at least three separate experiments per cell line. Statistical significance was determined by one-way ANOVA: MDA-MB-231 (*F*(3, 56) = 242.8, *p* < 0.001), MDA-MB-468 (*F*(3, 24) = 5.84, *p* < 0.004), MCF-7 (*F*(3, 56) = 146.3, *p* < 0.001), HCC-1419 (*F*(3, 56) = 95.2, *p* < 0.001), and SKBR-3 (*F*(3, 16) = 5.94, *p* < 0.006), followed by the post hoc Tukey. Error bars show the standard error of the mean.

Five human breast cancer cell lines, MDA-MB-231, MDA-MB-468, MCF-7, SKBR-3, and HCC-1419, were treated either with Th1 cytokines (IFN-*γ* and TNF-*α*), sunitinib, or both. The addition of Alamar Blue dye, which is reduced by metabolically active cells (inducing color change), was used to determine the impact of individual and combined treatment on the cells (lower optical density corresponds to higher metabolic activity). Treatment with both Th1 cytokines and sunitinib resulted in a greater degree of metabolic suppression than either treatment alone for all 5 tested lines (Figure 1; *p* < 0.05). Isobole analysis (supplemental figure 1) appeared to indicate synergy between drug and cytokines.

### 3.2. Combination of Th1 Cytokines and Sunitinib Maximizes Cell Death

Alamar Blue assay detects suppression of metabolic activity in cells. However, it does not prove that any observed suppression in metabolism is a consequence of actual cell death. To confirm cell death, vital staining with Trypan Blue staining was performed. Breast cancer cell lines were exposed to either Th1 cytokines, sunitinib, or both for 72 hours, or left untreated (No Rx). Microscopic examination of cells suggested considerable cell death; all treatment groups displayed fewer intact cells, high levels of subcellular debris, with remaining viable cells exhibiting altered morphological features (not shown). Cells that were exposed to both treatments seemed to be most affected.

**Figure 2:**
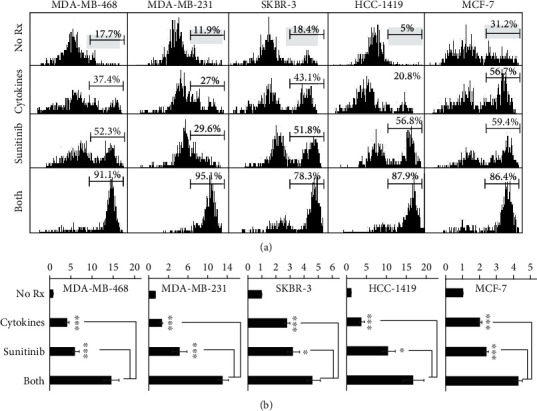
Trypan Blue retention maximized by dual treatment with sunitinib and Th1 cytokines. MDA-MB-468, MDA-MB-231, SKBR-3, HCC-1419, and MCF-7 cells were seeded at 5 × 10^4^ per mL and were left either untreated (No Rx) or treated with Th1 cytokines (5 ng/mL), sunitinib (10 *μ*M), or sunitinib and Th1 cytokines (both) for 72 hours. Cells were harvested and stained with Trypan Blue dye, after which dye uptake was detected via its fluorescent properties via flow cytometry using a 642 nm excitation laser. (a) Histogram analysis of representative experiment for each cell line. The gated region represents the percentage of Trypan Blue-retaining (dead) cells. (b) Data represent the summary analysis of mean channel fluorescent index of treated, stained cells from at least three separate experiments per cell line. Statistical significance was determined by one-way ANOVA: MDA-MB-231 (*F*(3, 56) = 46.1, *p* < 0.001), MDA-MB-468 (*F*(3, 52) = 33.1, *p* < 0.001), MCF-7 (*F*(3, 28) = 91.5, *p* < 0.001), HCC-1419 (*F*(3, 28) = 19.1, *p* < 0.001), and SKBR-3 (*F*(3, 24) = 22.4, *p* < 0.001), followed by the post hoc Tukey test. Error bars represent the standard error of the mean.

These visual observations were confirmed when the remaining cells were harvested and stained with Trypan Blue, which is excluded from living cells but retained in dead ones, imparting a strong fluorescent signal which can be assessed via flow cytometry. For the five tested cell lines, flow cytometric data of cellular fluorescence for each treatment group is displayed in histogram form (Figure 2(a)). During analysis, a region was established to distinguish low-staining (living) from high-staining (dead) cells, allowing a percentage cell death assignment for all treatment groups. For example, in one representative experiment MDA-MB-468, cell death was shown to be the highest with combined treatment: untreated (17.7%), Th1 cytokines (37.4%), sunitinib (52.3%), and both treatment (91.1%). MDA-MB-231 showed similar results: untreated (11.9%), Th1 cytokines (27%), sunitinib (29.6%), and both (95.1%). For SKBR-3 cells, 8.4% dead for untreated, 43.1% for Th1 cytokines, 51.8% for sunitinib, and 78.3% for both treatments. HCC-1419 cell death went from 5% untreated, 20.8% Th1 cytokines, 56.8% sunitinib, and 87.9% for both treatment combinations. Similar results were found for estrogen receptor-positive cell lines, MCF-7 with 31.2% dead in untreated, 56.7% Th1 cytokines, 59.4% sunitinib, and 86.4% in both treatment combinations.

We also analyzed the flow cytometry data by differences in overall fluorescent intensity (mean channel fluorescence index). Composite data from at least three separate experiments per cell line are illustrated in Figure 2(b). For all breast cancer cell lines tested, the combination of both Th1 cytokines plus sunitinib led to significantly greater uptake of fluorescent dye (*p* < 0.05) than either treatment alone. Taken together, these data demonstrated that the combination of Th1 cytokines plus sunitinib significantly increases cell death over either treatment alone.

### 3.3. Th1 Cytokines Combined with Sunitinib Potentiate the Expression of Cellular Markers Associated with Apoptosis

**Figure 3:**
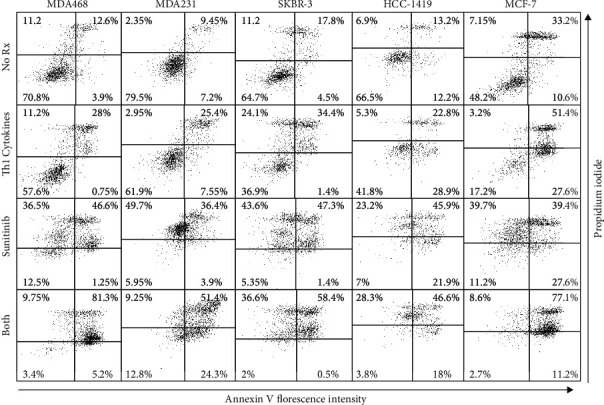
Combination of Th1 cytokines and sunitinib maximizes markers of apoptosis in breast cancer cell lines. MDA-MB-468, MDA-MB-231, SKBR-3, HCC-1419, and MCF-7 cells were seeded at 5 × 10^4^ per mL and were left either untreated (No Rx) or treated with Th1 cytokines (5 ng/mL), sunitinib (10 *μ*M), or sunitinib and Th1 cytokines (both) for 72 hours. Cells were then harvested and stained with FITC-Annexin V and propidium iodide (PI), and stain uptake was assessed by flow cytometry. Two-color dot plot analysis displays the percentage of cells in each quadrant. Double-stained cells (upper right quadrants) indicate late apoptotic events. Dot plots shown are from one representative experiment of three total showing similar results.

To determine if the observed cell death occurred through an apoptotic mechanism, a number of apoptosis-associated markers were examined. First, treated breast cancer cell lines were stained with FITC-Annexin V/propidium iodide (PI) and analyzed by flow cytometry (Figure 3). Cells in the late stages of apoptosis stain are simultaneously positive with both agents. For the MDA-MB-468 line, the proportion of cells that stained brightly for both Annexin V and PI was as follows: untreated 12.6%, Th1 cytokines 28%, sunitinib 46.6%, and both treatment 81.3%. For MDA-MB-231 cell lines, these values were untreated 9.45%, Th1 cytokines 25.4%, sunitinib 36.4%, and both treatment combinations 51.4%. For SKBR-3 cell lines, these values were 17.8% (untreated), 34.7% (Th1 cytokines), 47.3% (sunitinib), and 58.4% (both treatments). For HCC-1419 cell lines, these values were 17% (untreated), 22% (Th1 cytokines), 22.2% (sunitinib), and 34.8% (both treatments). Similarly, estrogen-positive cell lines MCF-7 represented 33% (untreated), 51.4% (Th1 cytokines), 39.4% (sunitinib), and 77.1% (both treatments). These results demonstrate that dual treatment of breast cancer cell lines with Th1 cytokines and sunitinib resulted in enhanced apoptotic cell death than either treatment alone.

**Figure 4:**
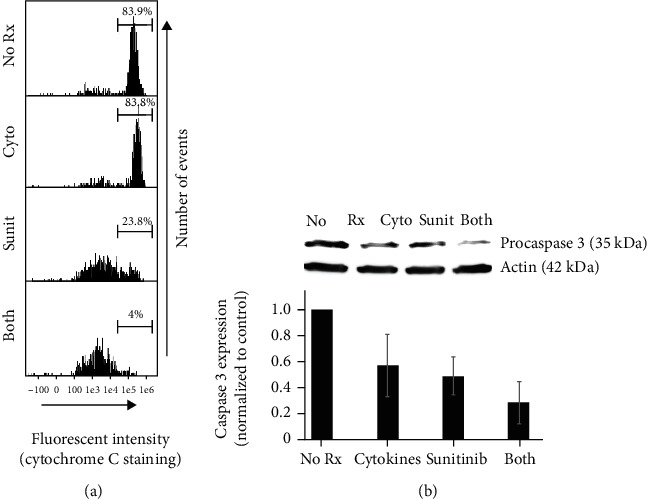
Sunitinib and Th1 cytokine treatment maximizes cytochrome C release from mitochondria and degradation of PARP. SKBR-3 cells were seeded at 5 × 10^4^ per mL and were left untreated (No Rx) or treated with Th1 cytokines (5 ng/mL each), sunitinib (10 *μ*M), or sunitinib and Th1 cytokines (both) for 72 hours. (a) Cells were harvested, permeabilized, and stained first with anti-cytochrome C antibodies, followed by an APC-labeled secondary antibody. Cells were then analyzed by flow cytometry using a 642 nm excitation laser. Histograms display a single representative experiment of cytochrome C expression in SKBR-3 cell lines out of four total experiments with similar results. (b) Treated cells were harvested and extracted in RIPA buffer in the presence of protease and phosphatase inhibitors. Protein lysate (30 *μ*g/well) was separated on a 4-15% SDS gel and electrotransferred onto nitrocellulose membranes. Blots were then probed with anti-PARP (116 kDa) and anti-actin (loading control; 42 kDa) and bands visualized using chemiluminescence substrate with the Fuji LAS 3000 detection system. Illustrated blot representative of 3 separate experiments with similar results.

We next turned our attention toward the loss of cytochrome C from mitochondria for representative cell line SKBR-3. Treated SKBR-3 were harvested, permeabilized (in a solution containing KCl and digitonin), and stained with anti-cytochrome C antibody. The permeabilizing solution disrupts plasma membrane integrity, allowing cytochrome C released from the mitochondria to leach out of the cell. However, the digitonin in the buffer does not compromise mitochondrial membranes, allowing for retention and staining of cytochrome C in these organelles. This difference allows only cytochrome C associated with the mitochondria to be measured [14]. For the SKBR-3 cell lines, flow cytometric data of cellular fluorescence of each treatment group is displayed in histogram form (Figure 4(a)). For this analysis, a region was defined to distinguish low-staining (loss of cytochrome C) from high-staining (cytochrome C retained in mitochondria) cells, allowing a determination of the percentage of cells retaining mitochondrial-associated cytochrome C. For example, in one representative experiment of SKBR-3, cytochrome C stain was the lowest with both treatments: no treatment (83.9%), Th1 cytokines (83.8%), sunitinib (23.8%), and both treatment combination (4%) (Figure 4(a)). Similar results were observed in additional trials.

Finally, we sought to indirectly assess the activity of executioner caspase 3 by examining the levels of one of its downstream targets, PARP, in treated SKBR-3 cells via Western blot analysis. Although single agents seemed to have little effect on PARP, the combination of sunitinib and Th1 cytokines appeared to display a trend toward loss of PARP protein in experimental trials (Figure 4(b)).

### 3.4. Th1 Cytokines Combined with Sunitinib Minimize the Expression of Surface HER-2 Compared with Single Treatments

**Figure 5:**
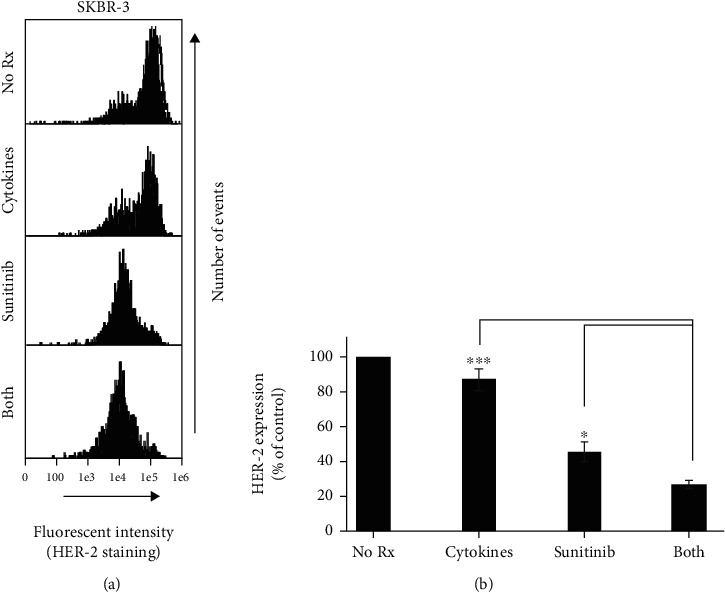
Dual treatment significantly suppresses HER-2 expression compared with single treatments. SKBR-3 cells were seeded at 5 × 10^4^ per mL and were left either untreated (No Rx) or treated with Th1 cytokines (5 ng/mL), sunitinib (10 *μ*M), or sunitinib and Th1 cytokines (both) for 72 hours. Cells were harvested and stained with APC-labeled anti-HER-2 antibody and evaluated by flow cytometry using a 642 nm excitation laser. (a) Histogram analysis of HER-2 expression from a single representative experiment. (b) Composite data analysis of mean channel fluorescence from three separate experiments for each treatment group. Statistical significance was determined by one-way ANOVA: (*F*(3, 20) = 65.7, *p* < 0.001) followed by Tukey post hoc analysis. Significance was set at ^∗∗∗^*p* < 0.001, ^∗∗^*p* < 0.01, and ^∗^*p* < 0.05. Error bars represent the standard error of the mean.

Previous studies had shown that Th1 cytokine treatment could lead to lower levels of HER-2 expression in some cell lines [4]. To determine whether the addition of sunitinib maximized HER-2 loss, surface HER-2 levels were examined in the SKBR-3 cell line. At 72 hours, posttreatment cells were harvested and stained with HER-2 antibody and analyzed by flow cytometry with results from a single representative experiment displayed in histogram form (Figure 5(a)), and composite data from 3 separate experiments analyzed by relative fluorescent intensity (Figure 5(b)). Histogram data reveal suppressed HER-2 expression, which is maximally achieved when SKBR-3 cells were exposed to both Th1 cytokines and sunitinib. Analysis of mean channel fluorescence from three separate experiments verified that the differences seen with dual treatments were significant over single treatments (Figure 5(b); *p* < 0.05).

### 3.5. Sunitinib Profoundly Suppresses IFN-*γ* Secretion by T Lymphocytes

**Figure 6:**
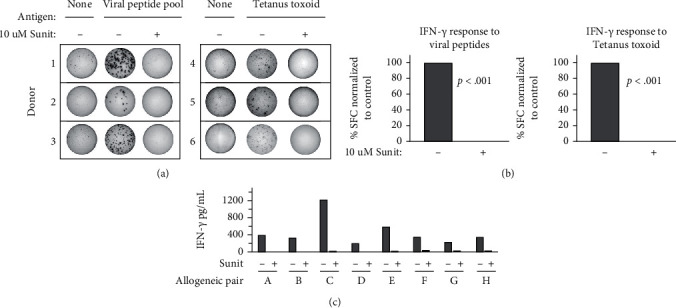
Sunitinib suppresses interferon-gamma secretion by stimulated T cells. (a) Example IFN-*γ* ELISPOT wells from individual healthy donor PBMCs stimulated with a mixture of common viral peptide recall antigens (left panel) or tetanus toxoid (right panel) in the presence or absence of sunitinib (“sunit”; 10 *μ*M). (b) Composite analysis of each of the 3 donors for viral recall peptides (left panel) and 3 donors for tetanus toxoid (right panel). Statistical analysis by one-way ANOVA indicated a significant difference between sunitinib-treated and sunitinib-untreated groups. (c) IFN-*γ* ELISA analysis of 72-hour culture supernatants from 8 unique allogeneic MLRs (A–H) where activated dendritic cells (DC) and lymphocyte-rich elutriation fractions were cocultured at 1 : 40 stimulator : responder ratios in the presence or absence of 10 *μ*M sunitinib.

Since sunitinib is a relatively broad-spectrum receptor tyrosine kinase inhibitor, we were concerned that this drug may interfere with T cell function. We therefore performed IFN-*γ* ELISPOT assays to check recall responses against a pool of common viral peptide antigens (Figures 6(a) and 6(b), left panels) as well as tetanus toxoid (Figures 6(a) and 6(b), right panels). We also performed allogeneic MLRs using activated human DCs as stimulators and lymphocyte-enriched white blood cell fractions from MHC-unmatched individuals as responder cells (Figure 6(c)). These cocultures were performed in the presence or absence of the same concentration of sunitinib used in the preceding studies on cancer cell lines. As can be seen in both sample ELISPOT wells (Figure 6(a)) and composite analysis of 3 separate donors for each antigen set (Figure 6(b)), the presence of sunitinib profoundly suppressed IFN-*γ* production. Likewise, across 8 unique allogenic pairings of stimulator DCs and responder T cells, almost complete suppression of IFN-*γ* production by sunitinib was observed (Figure 6(c)).

### 3.6. Therapy with Sunitinib plus Recombinant Interferon-Gamma Suppresses Tumor Growth in an Orthotopic Murine Model of HER-2^pos^ Breast Cancer

**Figure 7:**
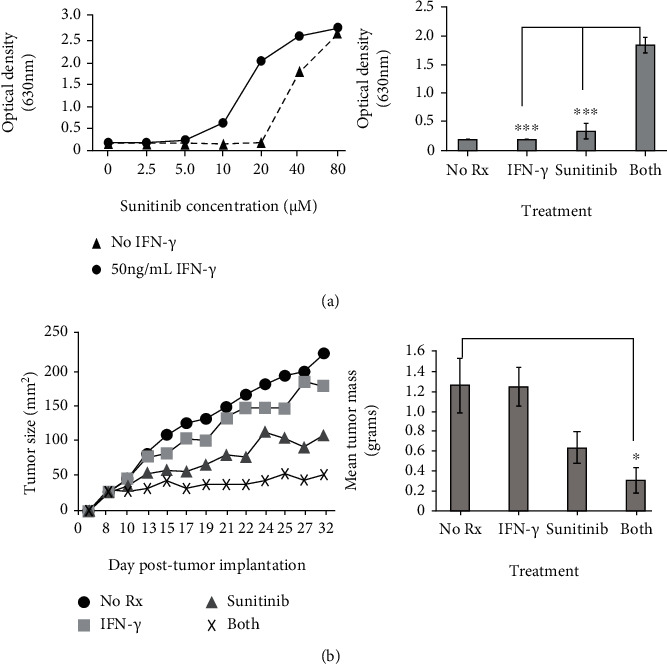
Combination therapy with sunitinib and recombinant IFN-*γ* suppresses tumor growth in an orthotopic murine model of HER-2^pos^ breast cancer. (a) Rat HER-2 (neu)-transgenic TUBO breast carcinoma cells were cultured with increasing concentrations (0-80 *μ*M) of sunitinib in the presence (circles) or absence (triangles) of 50 ng/mL recombinant murine IFN-*γ*. After 72 hours of treatment, metabolic activity was measured via the Alamar Blue assay (left panel). Right panel displays the statistical analysis of differences in dye metabolism between treatment groups at the optimal drug concentration of 20 *μ*M. (b) TUBO cells (2.5 × 10^5^) were implanted into the fat pad of the breast of female balb/c mice. Seven days later, therapy commenced consisting of i.p. administration of IFN-*γ* (10 *μ*g/mouse), sunitinib (1 mg/mouse), both treatments, or mice left untreated (control). Drug and cytokine therapy was supplied in 3 cycles (days 7-10, 16-18, and 24-25). Tumors were measured periodically using calipers to determine growth kinetics (left panel). On day 32, mice were sacrificed, tumors were excised and weighed, and average tumor mass for each treatment group was determined (right panel).

Because we demonstrated the capacity of sunitinib to suppress IFN-*γ* production by cultured T lymphocytes, it was concluded that it would be potentially challenging to combine this drug with an active immunotherapy regimen such as vaccination, as it would likely interfere with critical T cell functions. We therefore sought to design a potentially translatable therapy that bypasses the need for antigen-specific T cells by supplying recombinant IFN-*γ* along with drug. Since the preceding experiments in this study used two Th1 cytokines (TNF-*α* plus IFN-*γ*) combined with sunitinib, we wanted to first test whether IFN-*γ* only could substantially enhance sunitinib effects. Therefore, murine TUBO breast carcinoma cells were incubated with increasing concentrations of sunitinib in the presence or absence of murine recombinant IFN-*γ* (Figure 7(a), left panel). Statistical analysis of Alamar Blue assay data at the apparently optimal concentration of 20 *μ*g/mL sunitinib demonstrated a significant enhancement of suppression of metabolic activity in the dual treatment group compared with single treatments (Figure 7(a), right panel), indicating that TNF-*α* was not absolutely necessary for enhancing the activity of sunitinib.

Therefore, mice were implanted in the fat pad of one of the right breasts with TUBO breast carcinoma cells and were subsequently divided into 4 treatment groups (6 mice per group). When the tumors were palpable (day 7), therapy commenced consisting of IFN-*γ* (10 *μ*g/dose), sunitinib (1 mg/dose), and both agents simultaneously, or alternatively, mice were left untreated as controls. Tumors were measured periodically using calipers, and on day 32, mice were sacrificed, and tumors were carefully excised, trimmed of extraneous tissues, and weighed. Growth kinetics showed a little suppressive effect for IFN-*γ* alone, moderate suppression of growth for sunitinib alone, and almost total retardation of progressive growth in the dual-treatment group (Figure 7(b), left panel). Average tumor masses for each group showed the same pattern, with statistical analysis revealing that the only treatment significantly different from untreated control was the dual treatment group (IFN-*γ* plus sunitinib, *p* < 05); single treatments were not significantly different from control (Figure 7(b), right panel).

## 4. Discussion

Th1 cytokines IFN-*γ* and TNF-*α* have been known to induce apoptosis and senescence in various cancer cell lines [4, 5, 16] while suppressing the expression of a number of growth factor receptors such as HER-2 and HER-3 [4]. Similarly, sunitinib inhibits the activity of multiple RTKs [17]. Therefore, we anticipated that the combination of Th1 cytokines with sunitinib would work cooperatively to rob cancer cells of multiple important growth and survival signals, while simultaneously providing proapoptotic signals, thereby enhancing breast cancer cell death. For these studies, we examined HER-2^pos^/ER^pos^ (MCF-7), HER-2^pos^/ER^neg^ (SKBR-3 and HCC-1419), and triple-negative (MDA-MB-468 and MDA-MB-231) cell lines. In all cell lines, the combination of sunitinib and Th1 cytokines effectively suppressed metabolic activity (Figure 1) and induced cell death (Figure 2), through an apparent apoptotic mechanism (Figures 3 and 4). We further examined the expression level of growth factor receptor HER-2 in SKBR-3 cell lines and discovered that the combination of both sunitinib and Th1 cytokines led to significantly suppressed surface expression (*p* < 0.05).

We also encountered an initially unexpected difficulty during these studies arising from the fact that the drug sunitinib appeared to possess fluorescent properties persistent enough, even after washing of treated, harvested cells, to interfere with many of our commonly used fluorescent assays that rely upon excitation with the 488 nm laser and detection in some channels such as those used for the fluorochrome FITC and TMRE dye, the latter which we routinely use to measure mitochondrial membrane polarization (a property usually lost during apoptotic cell death). However, upon review of the recent literature, we found that this phenomenon of sunitinib fluorescence had in fact been observed previously [18]. In some cases, we were able to work around this limitation by substituting fluorochromes. For example, we exchanged FITC-labeled Annexin V with an APC conjugate that was excited by the 642 nm laser and detected in a channel far removed from the emission spectrum of sunitinib. For TMRE, however, there was no substitute, and we thus could not perform mitochondrial membrane polarization assays when sunitinib was used.

We did, however, examine a different aspect of mitochondrial function associated with apoptosis. One of the hallmarks for apoptotic cell death is the loss of cytochrome C from the mitochondria [14]. We measured cytochrome C levels in SKBR-3 cell lines by flow cytometry using the appropriate fluorochrome-labeled antibodies so as to avoid the problem of interference by the drug. As expected, cytochrome C was the lowest in cells treated with both Th1 cytokines and sunitinib (Figure 4). We were somewhat surprised, however, to observe that Th1 cytokine treatment alone did not result in an observable decrease in cytochrome C levels, given the fact that this treatment showed evidence of moderate cell death by all other criteria examined in these studies. To the point, TNF-*α*, one of the Th1 cytokines used, is known to bind directly to receptors involved in initiating the extrinsic apoptotic pathway [19]. TNF-*α* binds to a TNF superfamily receptor, and adaptor proteins such as TRADD (tumor necrosis factor receptor type 1-associated death domain protein) and FADD (Fas-associated protein with death domain) associated with the receptor [20]. This interaction leads to the formation of death-inducing signaling complex that cleaves procaspase 8, leading to activation of Bid (proapoptotic signal), which is known to cause the release of cytochrome C from mitochondria. However, despite the unexpected weakness of Th1 cytokines alone for inducing cytochrome C loss, when combined with sunitinib, cytochrome C loss was dramatically increased over drug alone.

Because each malignant tumor is unique, with its own set of mutations and dysregulated genes, most established cancer cell lines can be expected to behave somewhat differently with individual treatments; nonetheless, we found that sunitinib worked very well when combined with Th1 cytokines in a set of phenotypically diverse lines which included hormone receptor-positive, HER-2-positive, and triple-negative cells. This is likely due in part to the general lack of selectivity in sunitinib's capacity to antagonize the activity of multiple tyrosine kinases. Although much of sunitinib's activity has been ascribed to targets other than the malignant cells themselves (e.g., tumor vasculature [21] and myeloid-derived suppressor cells [22]), direct effects have also been observed. For example, in renal cell carcinoma, sunitinib has been shown to suppress the activation of STAT3 [23], a nuclear transcription factor considered critical for promoting proliferation, resistance to apoptosis, and tumorigenesis [24]. Interestingly, IFN-*γ* is known to promote the activation of STAT1, which works in opposition to STAT3 by suppressing cell proliferation and promoting apoptosis [25]. It seems likely that the cooperative effects of Th1 cytokines and sunitinib involve the simultaneous activation of STAT1 and suppression of STAT3.

Although these in vitro studies are highly encouraging, combining such drugs with Th1-polarizing vaccination has potential limitations that must be considered, since many of the signaling pathways in cancer cells potentially suppressed by inhibitors of receptor tyrosine kinases are also critical in immune system cells. Therefore, immune system function may be altered in unexpected ways. For example, studies by others have shown that sunitinib treatment for patients with renal cell cancer was associated with an increase in frequency of T cells producing IFN-*γ* (i.e., Th1 cells) and a decrease in T cells producing IL-4 (i.e., Th2 cells), which reverses the unfavorable Th1/Th2 ratio characteristic in this disease state. This shift is critical for generating an effective antitumor immune response [26]. In these renal cancer cells, T regulatory cells (which favor tumor growth) are elevated, which was shown to be reduced with sunitinib treatment. In addition, renal cell carcinoma patients treated with sunitinib demonstrated a reduction of myeloid-derived suppressor cells [22], which are also considered to promote tumor survival and growth. On the other hand, patients with metastatic clear cell renal cancer that were treated with sunitinib showed an overall depressed level of CD3^pos^ and CD4^pos^ T cells [27]. Whereas these preceding studies present a mixed bag of alterations that may be expected in some cases to promote, and others to attenuate antitumor immunity, it should be noted that all of these represent changes that were observed in immune system cells after a course of drug was supplied in vivo. They did not actually measure cell activity while under the direct influence of the drug. In contrast, relatively few studies have observed T cell function in the presence of tyrosine kinase inhibitor drugs. One recent study [28] did compare a panel of several tyrosine kinase inhibitor drugs for T cell suppressive activity and found the drug axitinib to be nonsuppressive relative to sunitinib. Our own in vitro studies seemed to indicate that sunitinib, at concentrations identical to those promoting tumor cell apoptosis in conjunction with recombinant cytokines, also displayed a potent capacity to nearly ablate antigen-specific IFN-*γ* secretion in both the allogenic MLR and to specific protein recall antigens (Figure 6). This observation poses interesting questions regarding unexplored limitations not only of sunitinib but also of other targeted drugs that have been developed as anticancer agents. These may profoundly suppress defined aspects of immunity while they are present in the body of the treated subject and may actually undermine substantial portions of their potential effectiveness that rely on intact immune function.

A final example of the complicated relationship between sunitinib's anticancer and immunomodulatory properties was demonstrated in a murine model by Jaini and coworkers [29]. Here, mice bearing 4T1 mammary carcinoma cells were vaccinated with a recombinant tumor antigen, lactalbumin, in CFA adjuvant. The experimental therapy also included the administration of sunitinib. Interestingly, when sunitinib was administered in combination with targeted immunotherapeutic vaccination, it affected the priming by the antigen-presenting cells (decrease in CD11b^pos^ and CD11c^pos^) and led to vaccination failure [29]. On the other hand, when sunitinib was administered after the priming phase of vaccination, there was an increase in vaccination-mediated immune response [29]. These latter studies make clear that relative timing of drug and immune-based therapy can also be an important issue. However, the role of Th1 cytokines as primary effectors of antitumor immunity is not known in this system. If they were, it is difficult to imagine how staggering vaccine and drug administration could be managed in such a way that both Th1 cytokine secretion by infiltrating lymphocytes and therapeutic concentrations of drug could be simultaneously achieved in tumor beds.

For our present studies, rather than attempting to stagger administration of active immunotherapy and sunitinib, we tried circumventing potential drug interference with immune responses by directly supplying recombinant IFN-*γ* concurrent with sunitinib administration. We omitted TNF-*α* entirely, because though this cytokine has been used with some success in immunotherapies involving isolated limb perfusion [30], there are known toxicities that limit its use systemically [31, 32], and thus, we did not consider systemic administration of TNF-*α* to be practically translatable. IFN-*γ*, on the other hand, has a better systemic safety profile and is currently approved and used clinically to treat chronic granulomatous disease [33]. We found that only combined, rather than single-agent therapy, suppressed tumor growth at statistically significant levels compared to untreated controls, suggesting this approach as a clinically translatable strategy for enhancing the activity of sunitinib, and perhaps other small-molecule-targeted agents. Because sunitinib is currently FDA-approved to treat renal cell carcinoma, we believe that testing recombinant IFN-*γ* in conjunction with this drug would be worthwhile, with the goal of increasing response rates with low risk of additional toxicity.

Interestingly, the idea of leveraging the effects of a targeted drug regimen by combining it with IFN-*γ* is an approach that we are already testing in a pilot clinical trial (NCT03112590) in the setting of locally advanced HER-2^pos^ breast cancer. For these subjects, the standard of care typically includes the combination of two anti-HER-2 antibodies (trastuzumab and pertuzumab) plus a taxane like paclitaxel, as well as a cytotoxic agent such as carboplatin. This trial substitutes IFN-*γ* for carboplatin. Although not yet completed, the midtrial results appear promising, with subjects experiencing nearly twice the pathologic complete response rates compared to historical controls receiving standard therapy, even while eliminating its most difficult-to-tolerate component (i.e., the carboplatin).

The studies presented in this manuscript demonstrate the proof-of-principle that a targeted small-molecule inhibitor of multiple receptor protein tyrosine kinases can work in conjunction with Th1 cytokines in vitro and in vivo to strongly enhance apoptosis in breast cancer cell lines of diverse phenotypes and suppress tumor growth in vivo for a murine model of HER-2^pos^ breast cancer. This suggests that certain targeted drugs can in theory be combined with promising anticancer vaccines that work largely through induction of strong Th1-dominated immune mechanisms, with the expectation that additive or synergistic effects can be achieved. However, they also expose the caveat that such drugs can have strong inhibitory effects on immune system cells that could render moot the cooperativity between drug and cytokines, which at least in some cases may be circumvented by administration of recombinant cytokines in lieu of vaccines or other cellular therapy. Further development of this general strategy will ideally involve the identification of more selective drugs that will similarly cooperate with Th1 cytokines to enhance tumor cell kill while simultaneously avoiding strong suppression of T cell activity.

## Data Availability

The data used to support the findings of this study are included within the article.

## Supplementary Materials

Supplementary Materials Supplemental Figure 1: for isobole analysis.

## References

[1] Disis M. L., Stanton S. E. (2018). Immunotherapy in breast cancer: an introduction. *Breast*.

[2] Czerniecki B., Koski G., Koldovsky U. (2007). Targeting HER-2/neu in early breast cancer development using dendritic cells with staged interleukin-12 burst secretion. *Cancer Research*.

[3] Sharma A., Koldovsky U., Xu S. (2012). HER-2 pulsed dendritic cell vaccine can eliminate HER-2 expression and impact ductal carcinoma in situ. *Cancer*.

[4] Namjoshi P., Showalter L., Czerniecki B., Koski G. (2016). T-helper 1-type cytokines induce apoptosis and loss of HER-family oncodriver expression in murine and human breast cancer cells. *Oncotarget*.

[5] Rosemblit C., Datta J., Lowenfeld L. (2018). Oncodriver inhibition and CD4(+) Th1 cytokines cooperate through Stat1 activation to induce tumor senescence and apoptosis in HER2+ and triple negative breast cancer: implications for combining immune and targeted therapies. *Oncotarget*.

[6] Lowenfeld L., Zaheer S., Oechsle C. (2017). Addition of anti-estrogen therapy to anti-HER2 dendritic cell vaccination improves regional nodal immune response and pathologic complete response rate in patients with ER(pos)/HER2(pos) early breast cancer. *Oncoimmunology*.

[7] Mendel D. B., Laird A. D., Xin X. (2003). In vivo antitumor activity of SU11248, a novel tyrosine kinase inhibitor targeting vascular endothelial growth factor and platelet-derived growth factor receptors: determination of a pharmacokinetic/pharmacodynamic relationship. *Clinical cancer research*.

[8] Morimoto A. M., Tan N., West K. (2004). Gene expression profiling of human colon xenograft tumors following treatment with SU11248, a multitargeted tyrosine kinase inhibitor. *Oncogene*.

[9] Bachelot T., Garcia-Saenz J. A., Verma S. (2014). Sunitinib in combination with trastuzumab for the treatment of advanced breast cancer: activity and safety results from a phase II study. *BMC Cancer*.

[10] Bergh J., Mariani G., Cardoso F. (2012). Clinical and pharmacokinetic study of sunitinib and docetaxel in women with advanced breast cancer. *Breast*.

[11] Crown J. P., Dieras V., Staroslawska E. (2013). Phase III trial of sunitinib in combination with capecitabine versus capecitabine monotherapy for the treatment of patients with pretreated metastatic breast cancer. *Journal of clinical oncology*.

[12] Showalter L. E., Oechsle C., Ghimirey N., Steele C., Czerniecki B. J., Koski G. K. (2019). Th1 cytokines sensitize HER-expressing breast cancer cells to lapatinib. *PLoS One*.

[13] Showalter L., Czerniecki B. J., Koski G. K. (2020). Th1 cytokines in conjunction with pharmacological Akt inhibition potentiate apoptosis of breast cancer cells in vitro and suppress tumor growth in vivo. *Oncotarget*.

[14] Christensen M. E., Jansen E. S., Sanchez W., Waterhouse N. J. (2013). Flow cytometry based assays for the measurement of apoptosis-associated mitochondrial membrane depolarisation and cytochrome c release. *Methods*.

[15] Czerniecki B. J., Carter C., Rivoltini L. (1997). Calcium ionophore-treated peripheral blood monocytes and dendritic cells rapidly display characteristics of activated dendritic cells. *Journal of Immunology*.

[16] Braumüller H. H. (2013). T-helper-1-cell cytokines drive cancer into senescence. *Nature*.

[17] Papaetis G. S., Syrigos K. N. (2009). Sunitinib: a multitargeted receptor tyrosine kinase inhibitor in the era of molecular cancer therapies. *BioDrugs*.

[18] Nowak-Sliwinska P., Weiss A., van Beijnum J. R. (2015). Photoactivation of lysosomally sequestered sunitinib after angiostatic treatment causes vascular occlusion and enhances tumor growth inhibition. *Cell Death & Disease*.

[19] Balachandran S., Kim C. N., Yeh W. C., Mak T. W., Bhalla K., Barber G. N. (1998). Activation of the dsRNA-dependent protein kinase, PKR, induces apoptosis through FADD-mediated death signaling. *The EMBO Journal*.

[20] Yuan C. H., Filippova M., Duerksen-Hughes P. (2012). Modulation of apoptotic pathways by human papillomaviruses (HPV): mechanisms and implications for therapy. *Viruses*.

[21] Schueneman A. J., Himmelfarb E., Geng L. (2003). SU11248 maintenance therapy prevents tumor regrowth after fractionated irradiation of murine tumor models. *Cancer Research*.

[22] Ko J. S., Zea A. H., Rini B. I. (2009). Sunitinib mediates reversal of myeloid-derived suppressor cell accumulation in renal cell carcinoma patients. *Clinical Cancer Research*.

[23] Xin H., Zhang C., Herrmann A., Du Y., Figlin R., Yu H. (2009). Sunitinib inhibition of Stat3 induces renal cell carcinoma tumor cell apoptosis and reduces immunosuppressive cells. *Cancer Research*.

[24] Grivennikov S., Karin E., Terzic J. (2009). IL-6 and Stat3 are required for survival of intestinal epithelial cells and development of colitis-associated cancer. *Cancer Cell*.

[25] Avalle L., Pensa S., Regis G., Novelli F., Poli V. (2012). STAT1 and STAT3 in tumorigenesis: a matter of balance. *Jakstat*.

[26] Finke J. H., Rini B., Ireland J. (2008). Sunitinib reverses type-1 immune suppression and decreases T-regulatory cells in renal cell carcinoma patients. *Clinical Cancer Research*.

[27] Powles T., Chowdhury S., Bower M. (2011). The effect of sunitinib on immune subsets in metastatic clear cell renal cancer. *Urologia Internationalis*.

[28] Stehle F., Schulz K., Fahldieck C. (2013). Reduced immunosuppressive properties of axitinib in comparison with other tyrosine kinase inhibitors. *The Journal of Biological Chemistry*.

[29] Jaini R., Rayman P., Cohen P. A., Finke J. H., Tuohy V. K. (2014). Combination of sunitinib with anti-tumor vaccination inhibits T cell priming and requires careful scheduling to achieve productive immunotherapy. *International Journal of Cancer*.

[30] Kahn J. O., Kaplan L. D., Volberding P. A. (1989). Intralesional recombinant tumor necrosis factor-alpha for AIDS-associated Kaposi’s sarcoma: a randomized, double-blind trial. *Journal of Acquired Immune Deficiency Syndromes*.

[31] Kimura K., Taguchi T., Urushizaki I. (1987). Phase I study of recombinant human tumor necrosis factor. *Cancer Chemotherapy and Pharmacology*.

[32] Muggia F. M., Brown T. D., Goodman P. J. (1992). High incidence of coagualopathy in phase II studies of recombinant tumor necrosis factor in advanced pancreatic and gastric cancers. *Anti-Cancer Drugs*.

[33] Ahlin A., Larfars G., Elinder G., Palmblad J., Gyllenhammar H. (1999). Gamma interferon treatment of patients with chronic granulomatous disease is associated with augmented production of nitric oxide by polymorphonuclear neutrophils. *Clinical and Diagnostic Laboratory Immunology*.

