# Supplementary material for: Sunitinib Combined with Th1 Cytokines Potentiates Apoptosis in Human Breast Cancer Cells and Suppresses Tumor Growth in a Murine Model of HER-2pos Breast Cancer
Source: Int J Breast Cancer. 2021 Apr 14;2021:8818393. doi: 10.1155/2021/8818393 (PMC8062178; doi:10.1155/2021/8818393)

## Slide 1
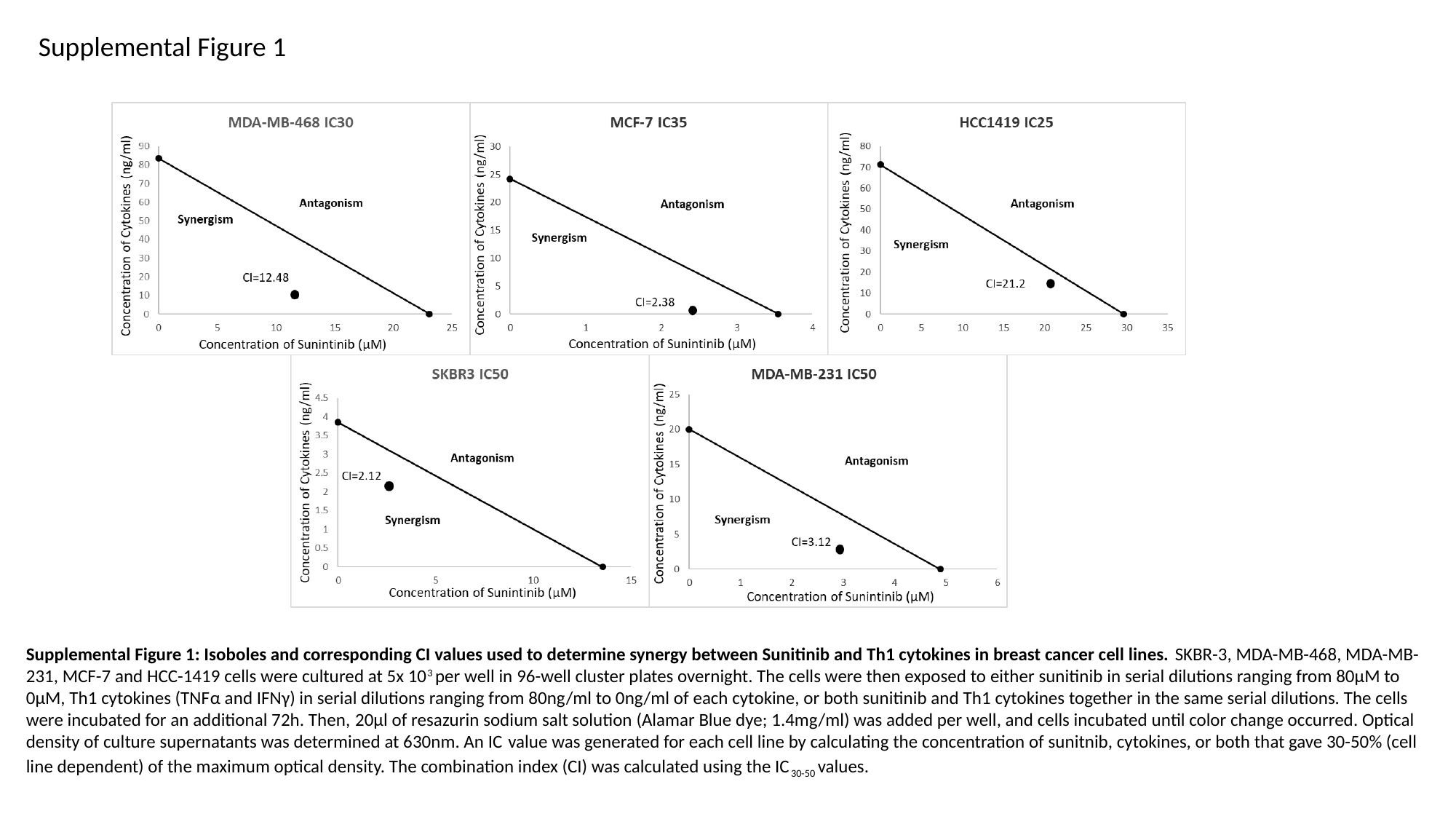

Supplemental Figure 1
Supplemental Figure 1: Isoboles and corresponding CI values used to determine synergy between Sunitinib and Th1 cytokines in breast cancer cell lines. SKBR-3, MDA-MB-468, MDA-MB-231, MCF-7 and HCC-1419 cells were cultured at 5x 10^3^ per well in 96-well cluster plates overnight. The cells were then exposed to either sunitinib in serial dilutions ranging from 80µM to 0µM, Th1 cytokines (TNFα and IFNγ) in serial dilutions ranging from 80ng/ml to 0ng/ml of each cytokine, or both sunitinib and Th1 cytokines together in the same serial dilutions. The cells were incubated for an additional 72h. Then, 20µl of resazurin sodium salt solution (Alamar Blue dye; 1.4mg/ml) was added per well, and cells incubated until color change occurred. Optical density of culture supernatants was determined at 630nm. An IC value was generated for each cell line by calculating the concentration of sunitnib, cytokines, or both that gave 30-50% (cell line dependent) of the maximum optical density. The combination index (CI) was calculated using the IC30-50 values.

## Slide 2
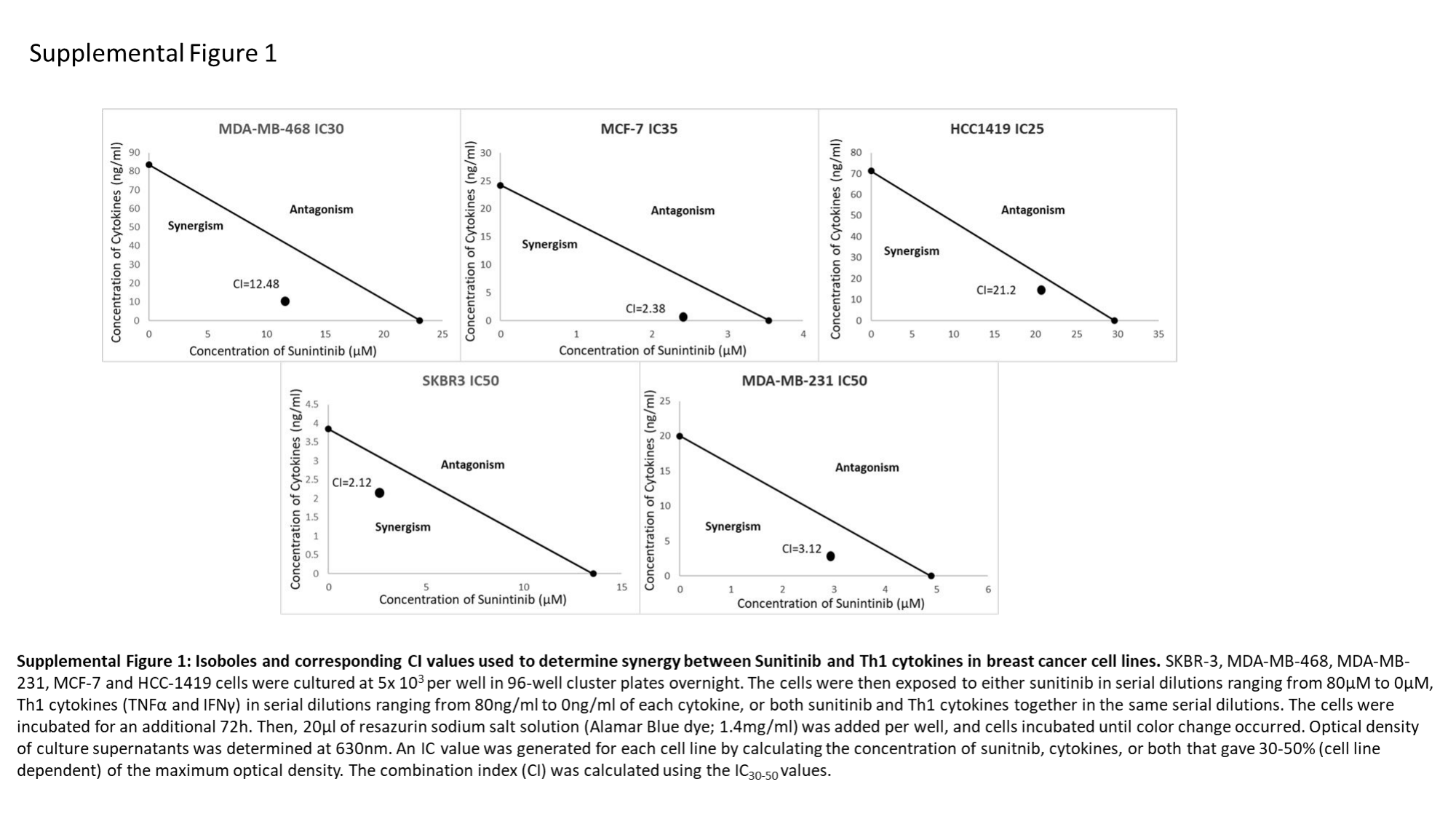

Supplement: Supplementary Materials — Supplemental Figure 1: for isobole analysis. [file 8818393.f1.pptx]
